# Three members of a peptide family are differentially distributed and elicit differential state-dependent responses in a pattern generator-effector system

**DOI:** 10.1152/jn.00850.2017

**Published:** 2018-01-31

**Authors:** Patsy S. Dickinson, Matthew K. Armstrong, Evyn S. Dickinson, Rebecca Fernandez, Alexandra Miller, Sovannarath Pong, Brian W. Powers, Alixander Pupo-Wiss, Meredith E. Stanhope, Patrick J. Walsh, Teerawat Wiwatpanit, Andrew E. Christie

**Affiliations:** ^1^Department of Biology, Bowdoin College, Brunswick, Maine; ^2^Békésy Laboratory of Neurobiology, Pacific Biosciences Research Center, School of Ocean and Earth Science and Technology, University of Hawaii at Manoa, Honolulu, Hawaii

**Keywords:** cardiac ganglion, central pattern generator, C-type allatostatin, *Homarus americanus*, *Manduca sexta*-type allatostatin, neurohormone, neuromodulator, neuropeptide, pericardial organ, PISCF-type allatostatin

## Abstract

C-type allatostatins (AST-Cs) are pleiotropic neuropeptides that are broadly conserved within arthropods; the presence of three AST-C isoforms, encoded by paralog genes, is common. However, these peptides are hypothesized to act through a single receptor, thereby exerting similar bioactivities within each species. We investigated this hypothesis in the American lobster, *Homarus americanus*, mapping the distributions of AST-C isoforms within relevant regions of the nervous system and digestive tract, and comparing their modulatory influences on the cardiac neuromuscular system. Immunohistochemistry showed that in the pericardial organ, a neuroendocrine release site, AST-C I and/or III and AST-C II are contained within distinct populations of release terminals. Moreover, AST-C I/III-like immunoreactivity was seen in midgut epithelial endocrine cells and the cardiac ganglion (CG), whereas AST-C II-like immunoreactivity was not seen in these tissues. These data suggest that AST-C I and/or III can modulate the CG both locally and hormonally; AST-C II likely acts on the CG solely as a hormonal modulator. Physiological studies demonstrated that all three AST-C isoforms can exert differential effects, including both increases and decreases, on contraction amplitude and frequency when perfused through the heart. However, in contrast to many state-dependent modulatory changes, the changes in contraction amplitude and frequency elicited by the AST-Cs were not functions of the baseline parameters. The responses to AST-C I and III, neither of which is COOH-terminally amidated, are more similar to one another than they are to the responses elicited by AST-C II, which is COOH-terminally amidated. These results suggest that the three AST-C isoforms are differentially distributed in the lobster nervous system/midgut and can elicit distinct behaviors from the cardiac neuromuscular system, with particular structural features, e.g., COOH-terminal amidation, likely important in determining the effects of the peptides.

**NEW & NOTEWORTHY** Multiple isoforms of many peptides exert similar effects on neural circuits. In this study we show that each of the three isoforms of C-type allatostatin (AST-C) can exert differential effects, including both increases and decreases in contraction amplitude and frequency, on the lobster cardiac neuromuscular system. The distribution of effects elicited by the nonamidated isoforms AST-C I and III are more similar to one another than to the effects of the amidated AST-C II.

## INTRODUCTION

The outputs of the pattern-generating networks responsible for controlling a wide range of rhythmic movements are remarkably flexible; this flexibility is due largely to modulation by amines and peptides. It is clear that such modulation is often state dependent (reviewed in Marder et al. 2014); thus the same modulator can produce stronger or weaker effects depending on the initial state of the system. One indicator of the “state” is often the baseline frequency or the baseline intensity of the motor output. In the crustacean stomatogastric nervous system (STNS), for example, excitatory inputs, including identified modulatory neurons [e.g., the anterior pyloric modulator (APM) and the modulatory proctolin neuron (MPN); Nagy and Dickinson 1983; Nusbaum and Marder 1989] as well as inhibitory inputs, such as bath-applied allatostatin peptides (e.g., Dickinson et al. 2009; Fu et al. 2007; Ma et al. 2009; Szabo et al. 2011) exert stronger effects when the initial pattern is weaker. In other cases, it is clear that interactions between different neuromodulators are critical in determining the effects of a given modulator. This is seen, for example, in the leech (Mesce et al. 2001; Mesce 2002), in which the simultaneous application of octopamine and serotonin inhibits swimming, whereas each amine applied on its own enhances swimming, and in the spiny lobster (Dickinson et al. 1997), in which the response of the cardiac sac pattern generator to the neuropeptide proctolin differs depending on its past history of exposure to another peptide, red pigment concentrating hormone (RPCH). Similarly, the effects of the neuropeptide allatotropin on the movements of the heart and aorta in several species of hemophagous insects are dependent on interactions with other modulators; this peptide increases the frequency of contractions, but only in the presence of serotonin (Sterkel et al. 2010; Villalobos-Sambucaro et al. 2015, 2016).

In the heart of the American lobster, *Homarus americanus*, the neuropeptide C-type allatostatin (pQIRYHQCYFNPISCF) exerts effects that are presumed to be state dependent, but the factors controlling the “state” are less evident (Wiwatpanit et al. 2012). In this case, the peptide most often causes a decrease in cardiac contraction frequency. At the same time, contraction amplitude decreases in the majority of lobsters; this decrease is due largely to a decrease in facilitation that results from the decreased frequency (Williams et al. 2013; Wiwatpanit et al. 2012). However, in some lobsters, the same peptide elicits an increase in contraction amplitude.

The C-type, or PISCF-type, allatostatins (AST-Cs) constitute a family of peptides that are related to the vertebrate somatostatins (Alzugaray et al. 2016; Auerswald et al. 2001; Birgül et al. 1999; Kreienkamp et al. 2002; Urlacher et al. 2016; Veenstra 2009). Initially identified in holometabolous insects, they are characterized by the carboxyl (COOH)-terminus −PISCF, as well as by a disulfide bridge between two internal cysteine residues (Stay and Tobe 2007). Early studies suggested the existence of two AST-C-like peptides, each encoded by a separate gene, which were hypothesized to be paralogs, in most members of the Arthropoda (Veenstra 2009). Subsequently, a third paralog was identified, so that it now appears there are three AST-C peptides in many arthropods (Dircksen et al. 2011; Veenstra 2016a). The precursor proteins for the three AST-C isoforms are thought to have arisen by triplication of the AST-C gene (Veenstra 2016a).

In crustaceans, two AST-C peptides, pQIRYHQCYFNPISCF (AST-C I; ortholog to AST-C; Veenstra 2016a) and SYWKQCAFNAVSCFamide (AST-C II; ortholog to AST-CCC; Veenstra 2016a) (disulfide bridging between the 2 cysteine residues in each peptide) were initially identified using a combination of transcriptomics and mass spectrometry (Dickinson et al. 2009; Gard et al. 2009; Ma et al. 2009; Stemmler et al. 2010; Veenstra 2009; Weaver and Audsley 2009); these two AST-Cs have been shown to be broadly, perhaps ubiquitously, conserved in members of the Decapoda (Dickinson et al. 2009; Stemmler et al. 2010). A third AST-C peptide, GNGDGRLYWRCYFNAVSCF (AST-C III; disulfide bridging between the 2 cysteines; ortholog to AST-CC; Veenstra 2016a) was more recently identified using transcriptomics (Christie et al. 2017; Veenstra 2015, 2016a, 2016b). This peptide has thus far been found in six decapod species, including *H. americanus* (Christie et al. 2017; Veenstra 2016b). The sequences of the *H. americanus* AST-Cs, including alternate nomenclatures from Veenstra (2016a), are shown in Table 1. Although studies of the functional roles served by AST-Cs in crustaceans are limited, both AST-C I and AST-C II have been shown to be bioactive in at least one member of the taxon (Dickinson et al. 2009; Ma et al. 2009; Wiwatpanit et al. 2012). For example, in the crab *Cancer borealis*, both AST-C I and AST-C II were reported to decrease the ongoing activity of the pyloric neural circuit (Ma et al. 2009), which is located within the STNS. The effects elicited on this system by the two peptides were essentially identical (Ma et al. 2009).

**Table 1.** Homarus AST-C isoforms

Peptide	Sequence	Veenstra Nomenclature
AST-C I	pQIRYHQCYFNPISCF	AST-C
AST-C II	SYWKQCAFNAVSCFamide	AST-CCC
AST-C III	GNGDGRLYWRCYFNAVSCF	AST-CC

Sequences of the three *Homarus* allatostatin-C (AST-C) peptides, with the nomenclature used in the present study and alternative nomenclature from Veenstra (2016a). Underlined cysteine (C) residues indicate a disulfide bond.

Despite the fact that at least five different somatostatin receptors have been identified in vertebrates (Barbieri et al. 2008, 2013; Patel 1999), only a single AST-C receptor has been identified in the majority of insect species studied (Kreienkamp et al. 2002; Mayoral et al. 2010). A notable exception is in the Diptera, including both *Drosophila melanogaster* (Kreienkamp et al. 2002) and the mosquito *Aedes aegypti* (Mayoral et al. 2010), where two AST-C receptors have been identified (Kreienkamp et al. 2002; Mayoral et al. 2010). Nonetheless, in those insects in which the functional effects of multiple AST-Cs have been examined (Audsley et al. 2013; Dong et al. 2017; Urlacher et al. 2016, 2017), it has been suggested that the peptides act on the single receptor that was identified, although potentially with different affinities (e.g., Urlacher et al. 2016). Interestingly, in one of the few crustaceans in which neuropeptide receptors have been predicted, *H. americanus*, three different AST-C receptors were recently identified using a transcriptome derived from mixed nervous system tissues (Christie et al. 2015).

In light of the facts that multiple putative AST-C receptors have been identified in the lobster nervous system (Christie et al. 2015) and that AST-C I elicits different effects on the cardiac neuromuscular system in different lobsters (Wiwatpanit et al. 2012), we were interested in determining whether the same is true for the other lobster AST-C peptides. Our data showed that all three AST-C peptides elicited both increases and decreases in heart contraction amplitude. This led us to ask whether the effects of all three peptides were always the same in an individual lobster; that is, if one isoform of AST-C elicits an increase in contraction amplitude, do the others likewise elicit increases in amplitude? Additionally, to determine whether the effects exerted by these peptides on the cardiac system are likely to be physiological, we explored the presence and distribution of the AST-C peptides in the cardiac ganglion (CG) and in endocrine/neuroendocrine tissues that might be responsible for providing AST-C to the cardiac neuromuscular system in the American lobster *H. americanus* via the circulatory system. To examine the distribution of these peptides, we used two antibodies that had been generated against AST-C peptides. These antibodies do not distinguish between AST-C I and AST-C III, neither of which is amidated, but they do distinguish between AST-C I/III and AST-C II, which is amidated (Christie et al. 2018). We used these antibodies to map the distributions of AST-C I/III and AST-C II in the eyestalk ganglia, including the X-organ-sinus gland (XO-SG) system, the pericardial organ (PO), the epithelial endocrine cells of the midgut and midgut cecum, and the CG. Taken collectively, our results suggest that these structurally related peptides, putatively encoded by paralog genes, are differentially distributed in the lobster nervous system and midgut and can elicit distinct behaviors from the cardiac neuromuscular system. Moreover, they suggest the possibility that particular structural features, e.g., COOH-terminal amidation, may be important in determining the specific effects of the peptide.

## MATERIALS AND METHODS

### Animals and Tissue Collection

American lobsters, *H. americanus* Milne-Edwards, were purchased from local suppliers in the Brunswick area of Maine and were maintained for up to 3 wk in recirculating natural seawater aquaria at 10–12°C. Animals were fed weekly with chopped shrimp and squid. For tissue collection, animals were cold-anesthetized in ice for 30–60 min, after which tissues (eyestalk ganglia, CG, POs, midgut, and midgut cecum) were collected via manual microdissection in chilled physiological saline (composition in mM: 479.12 NaCl, 12.74 KCl, 13.67 CaCl_2_, 20.00 MgSO_4_, 3.91 Na_2_SO_4_, 11.45 Trizma base, and 4.82 maleic acid; pH 7.45).

### Immunohistochemistry

#### Antibodies.

To map the distribution of the two nonamidated AST-C isoforms, AST-C I (pQIRYHQCYFNPISCF, ortholog to AST-C; Veenstra 2016a) and AST-C III (GNGDGRLYWRCYFNAVSCF, ortholog to AST-CC; Veenstra 2016a), we used a custom-produced (Lampire Biological Laboratories, Pipersville, PA) rabbit polyclonal antibody generated against the peptide AST-C I; this antibody was used at a final dilution of 1:5,000. Specificity tests, in which this antibody was preadsorbed with each of the three AST-C isoforms (see Christie et al. 2018) showed that it also cross-reacts with AST-C III, but not with AST-C II; thus we were unable to distinguish between the localization of AST-C I and AST-C III. For the detection of the amidated AST-C isoform AST-C II (SYWKQCAFNAVSCFamide, ortholog to AST-CCC; Veenstra 2016a), a custom-produced (Lampire Biological Laboratories) guinea pig polyclonal antibody was used at a final dilution of 1:8,000. Antibody preadsorptions with each of the three AST-C isoforms (see Christie et al. 2018) showed that this antibody is specific for AST-C II.

#### Whole mount immunohistochemistry.

For whole mount immunohistochemistry, tissues were fixed for 12–24 h at 4°C in a solution of 4% paraformaldehyde (EM grade, catalog no. 15710; Electron Microscopy Sciences; Hatfield, PA) in 0.1 M sodium phosphate buffer, pH 7.4 (P). Following fixation, tissues were rinsed 5 times at ~1-h intervals at room temperature (18–22°C) in P containing 0.3% Triton-X 100 (P-Triton), after which they were incubated for ~72 h at 4°C in primary antibody (or antibodies in the case of double-labeled preparations) diluted to final working concentration(s) (see above) in P-Triton containing 10% normal donkey serum (NDS; catalog no. 017-000-121; Jackson ImmunoResearch Laboratories, West Grove, PA). After incubation in primary antibody, tissues were rinsed 5 times at ~1-h intervals at room temperature in P-Triton and then incubated for 12–24 h at 4°C in one or two of the following secondary antibodies, diluted 1:300 in P-Triton containing 10% NDS: Alexa Fluor 488-conjugated AffiniPure donkey anti-rabbit IgG (catalog no. 711-545-152; Jackson ImmunoResearch Laboratories), Alexa Fluor 594-conjugated AffiniPure donkey anti-rabbit IgG (catalog no. 711-585-152; Jackson ImmunoResearch Laboratories), Alexa Fluor 647-conjugated AffiniPure donkey anti-rabbit IgG (catalog no. 711-605-152; Jackson ImmunoResearch Laboratories), Alexa Fluor 488-conjugated AffiniPure donkey anti-guinea pig IgG (catalog no. 706-545-148; Jackson ImmunoResearch Laboratories), Alexa Fluor 594-conjugated AffiniPure donkey anti-guinea pig IgG (catalog no. 706-585-148; Jackson ImmunoResearch Laboratories), Alexa Fluor 647-conjugated AffiniPure donkey anti-guinea pig IgG (catalog no. 706-605-148; Jackson ImmunoResearch Laboratories), Alexa Fluor 488-conjugated highly cross-adsorbed goat anti-guinea pig IgG (catalog no. A-11073; ThermoFisher Scientific, Carlsbad, CA), and Alexa Fluor 568-conjugated highly cross-adsorbed goat anti-guinea pig IgG (catalog no. A-11075; ThermoFisher Scientific). After incubation in secondary antibody, tissues were rinsed 5 times at ~1-h intervals at room temperature in P and then mounted between a glass microscope slide and coverslip in Vectashield mounting medium (catalog no. H-1000; Vector Laboratories, Burlingame, CA). Incubation in secondary antibody and subsequent processing was conducted in the dark; slides were stored at 4°C in the dark until examined for labeling.

#### Imaging.

Data were collected using an Olympus BX51 fluorescence microscope (Olympus America, Center Valley, PA) or a Leica TCS SP8 laser scanning confocal system mounted on a Leica DM6 CS upright digital research microscope (Leica Microsystems, Buffalo Grove, IL). Imaging on the Olympus BX51 system was done using a UPlanFI ×10 or UPlanFL N ×40 objective lens and manufacturer-supplied green fluorescence protein (GFP) or Texas red filter sets. On the Leica TCS SP8 system, imaging used either a HC PL APO ×10x or ×20 objective lens, with excitation/detection parameters adjusted/optimized using the system’s manufacturer-supplied hardware for the specific fluorochrome(s) present in a given preparation.

Digital images collected using the Leica confocal system were exported as .tiff files. The contrast and brightness of the confocal micrographs were adjusted to optimize the clarity of the printed images using Adobe Photoshop (Adobe Systems, San Jose, CA).

### Cardiac Physiology

To assess the cardiotropic effects of AST-C I, II, and III in *H. americanus*, the peptides were applied to the isolated whole heart, similarly to past studies (e.g., Dickinson et al. 2007, 2015a, 2015b; Stevens et al. 2008). Lobsters were anesthetized by packing in ice for 30–60 min, after which the posterior dorsal region of the carapace, which lies directly over the heart, and the underlying cardiac tissue were removed. This dissected region containing the heart was pinned through the carapace to the bottom of a small dish lined with Sylgard 184 (KR Anderson, Santa Clara, CA). The dorsal part of the heart remained attached to the carapace so that the extent to which it was stretched was identical to that in the intact animal. The posterior artery was cannulated with a short piece of polyethylene tubing extending into the heart, beyond the arterial valves, and was continuously perfused with chilled physiological saline at a flow rate of ~2.5 ml/min. A second perfusion line was directed across the top of the heart to help maintain temperature, which was monitored continuously and kept between 10° and 12°C with a Peltier temperature control system (CL100 bipolar temperature controller and SC-20 solution heater/cooler; Warner Instruments, Hamden, CT).

To record heart contractions, the anterior arteries were tied off with 6–0 suture silk and attached to a Grass FT03 force-displacement transducer (Astro-Med, West Warwick, RI) at an angle of ~30°–45° from horizontal, with an initial tension of 2 g. Preparations were allowed to stabilize for 1–2 h after the initial tension was applied before the first peptide application. The output of the transducer was amplified via an ETH-250 Bridge amplifier (CB Sciences, Dover, NH) and a Brownlee 410 instrumentation amplifier (Brownlee Precision, San Jose, CA) and recorded on a personal computer using a 1401 or Micro 1401 data acquisition board and Spike2 version 6 or 7 software (Cambridge Electronic Design, Cambridge, UK).

### Peptides

AST-C I, II, and III were custom synthesized by GenScript (Piscataway, NJ). Peptides were dissolved in deionized water at a concentration of 10^−3^ M and kept as a frozen stock solution at −20°C for use in physiological experiments. This peptide stock was diluted in chilled physiological saline to a final concentration of 10^−7^ M just before use. All three peptides were applied by perfusion through the cannulated artery. The three peptides were applied in random order, with ~1 h of saline wash between peptides, to allow for comparisons within the same preparation. Only preparations that returned to baseline activity after each peptide were used for analysis. No order effect was seen (data not shown).

### Data Analysis

Both heart rate and contraction amplitude were measured using the built-in functions of Spike2 and a script custom-written for this purpose. Data were further analyzed and graphed using Microsoft Excel and Prism 7 software (GraphPad Software, San Diego, CA). In this study, two contraction parameters, frequency (Hz) and amplitude (g), were used to assess the cardiotropic actions of the three AST-C peptides. The effects of the peptides on these parameters were measured as percent change from control levels to enable comparison of preparations with different baseline values. The percent changes from baseline for each parameter during peptide application were based on the mean values determined for the 200 s just before peptide application (control) and the mean of the 200 s at the peak of the peptide effect, 6–10 min after the onset of peptide application. Additionally, we examined the correlations of each pair of peptides using Pearson correlation. Because each peptide elicited a range of effects, we also used the Gaussian fit function of Prism 7 to fit a frequency histogram of the responses. To compare the distributions of effects among peptides, we used a Kolmogorov-Smirnov test (Prism 7) with a Bonferroni correction for multiple comparisons. All statistical comparisons were conducted using only those preparations in which all three peptides were applied and in which contraction parameters returned to approximately baseline levels after the perfusion of each peptide (*n* = 53). For all statistical analyses, a *P* value of 0.05 was used to indicate significance.

## RESULTS

### AST-C I/III- and AST-C II-Like Immunoreactivities Were Detected in Endocrine/Neuroendocrine Tissues and/or in the CG

#### Cardiac nervous system.

**Fig. 1.**
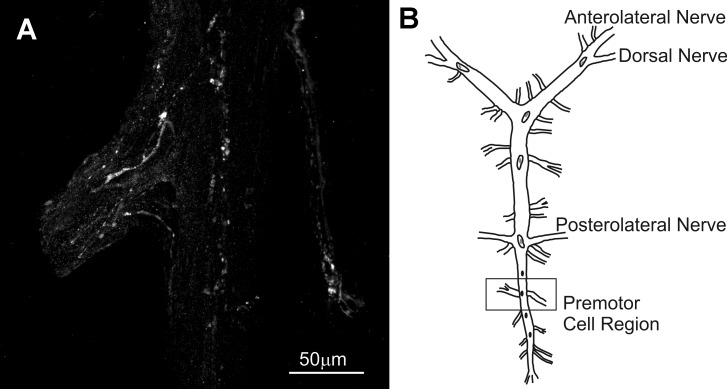
C-type allatostatin (AST-C) I/III-like immunoreactivity was present in the cardiac ganglion (CG). *A*: punctate nerve terminals, likely sites of peptide release, were located at several sites within the region of the CG, notably in regions in which the premotor neurons are located, suggesting that one or both of these peptides have access to these neurons. *B*: diagrammatic view of the CG; box indicates the region shown in *A*. This image is a maximum projection of 18 optical sections taken at 2-µm intervals.

No AST-C I/III- or AST-C II-like immunoreactivity was present in any of the somata in the CG (*n* = 7 preparations for each antibody). However, nerve terminals labeled by the AST-C I/III antibody were present in several regions of the CG in all preparations examined (Fig. 1). These terminals were superficially located within or just below the sheath of the CG; their origin remains unknown. No AST-C II-like immunoreactivity was detected in the CG sheath area.

#### Neuroendocrine organs.

In decapod species, the XO-SG system, located in the eyestalk, and the PO, located in the lateral pericardial chamber surrounding the heart, are two well-known neuroendocrine systems that contribute to the complement of peptide hormones present in the hemolymph (Christie 2011; Cooke and Sullivan 1982). No AST-C I/III- or AST-C II-like immunoreactivity was detected in the XO-SG (*n* = 8 preparations for each antibody; data not shown).

**Fig. 2.**
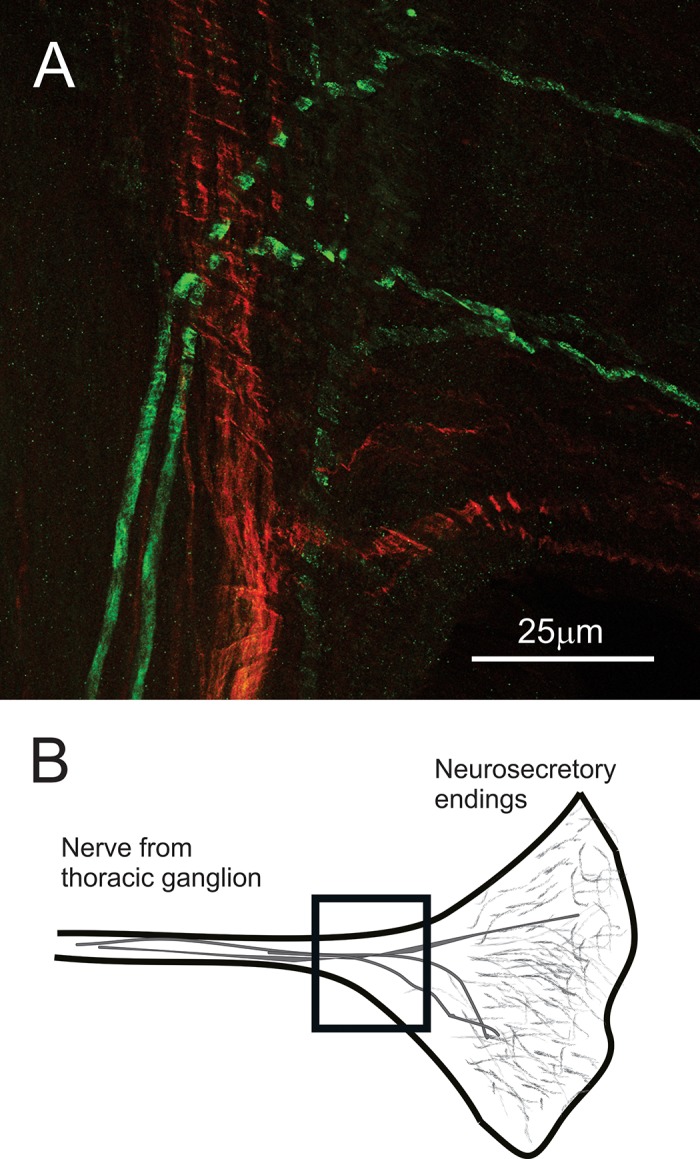
AST-C I/III-like immunoreactivity (green) was present in the pericardial organs (POs) of all lobsters examined; AST-C II-like immunoreactivity (red) was present in the POs of at least some lobsters (8 of 12). When present, immunoreactivity for both peptides appeared to be located in fibers as well as in punctate spots suggestive of release sites. However, there was no overlap of staining in any of the preparations observed (*n* = 10 double-labeled preparations). *A*: a single optical section taken from a double-labeled PO showing that AST-C I/III- and AST-II-like immunoreactivities are not colocalized in the PO. *B*: diagrammatic image of the PO showing the location (box) of the image shown in *A*.

In contrast to the XO-SG, both AST-C I/III- and AST-C II-like immunoreactivities were present in the PO (*n* = 12 preparations for each antibody; Fig. 2). Within the PO, the AST-C I/III-like immunoreactivity consisted of labeled axons and an extensive network of peripherally located fine neurites studded with small bleb-like terminals, which represent likely sites of peptide release. AST-C II-like immunoreactivity was much sparser but was nonetheless present in most preparations (*n* = 8 of 12). Again, both labeled axons and neurites associated with release terminals were present. Because the AST-C I/III antibody used in our study was generated in rabbit, whereas that used for the detection of AST-C II was made in guinea pig, we were able to directly examine the extent of colocalization of AST-C I/III and AST-C II by pairing the two antibodies in double-labeling studies. No colocalization was noted in any of the POs examined (*n* = 12 preparations, each with 2 POs; Fig. 2).

#### Endocrine cells of the midgut and midgut cecum.

**Fig. 3.**
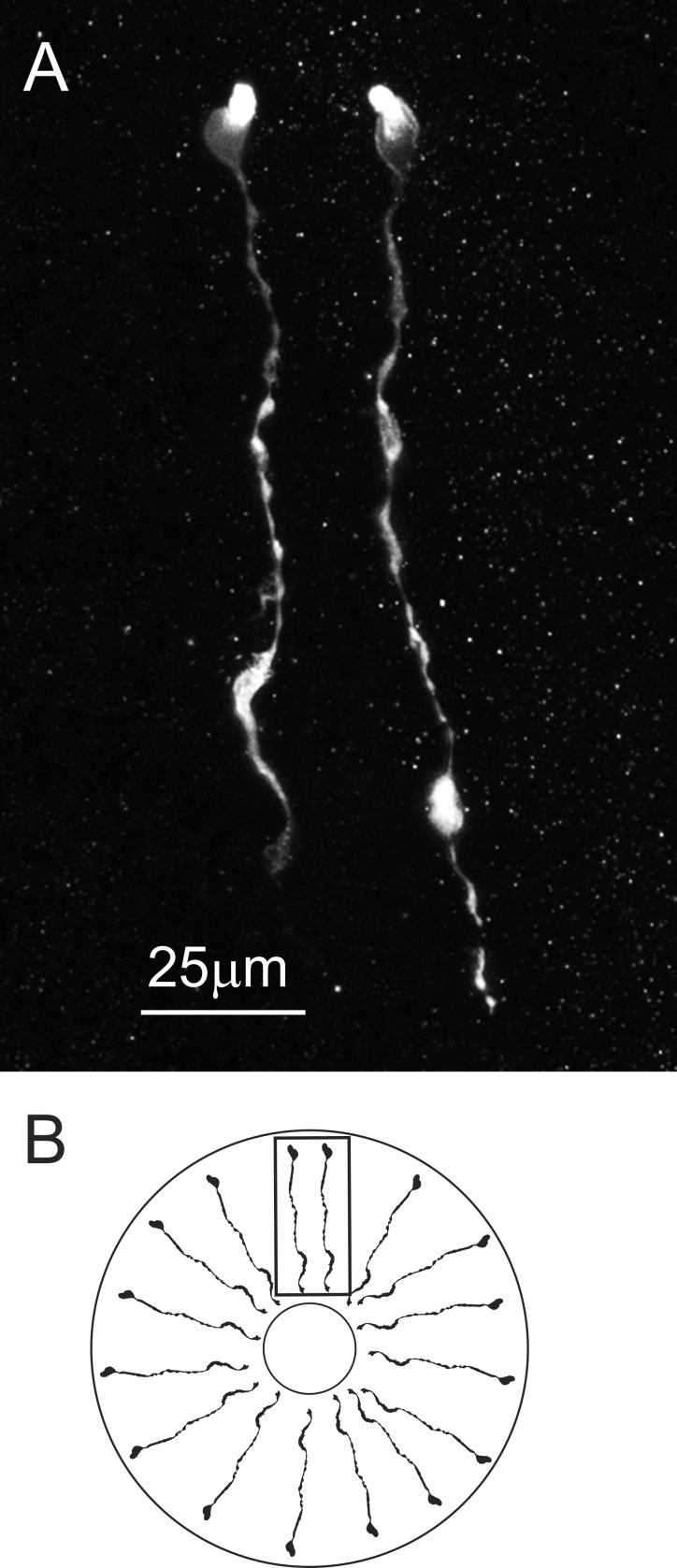
AST-C I/III-like immunoreactivity was present in epithelial endocrine cells in the posterior portion of the midgut. *A*: these immunopositive cells appear to span the epithelial layer and are morphologically similar to those previously described from the midgut of *Cancer* crabs (Christie et al. 2007). In the image shown, 2 AST-C I/III immunopositive cells are shown. This image is a maximum projection of 15 optical sections taken at 2-µm intervals. *B*: diagrammatic view of the midgut, showing the orientation of the AST-C I/III immunopositive midgut epithelial endocrine cells. Note that the drawing is not to scale: the lumen is shown much smaller than actual size. Box indicates orientation of image in *A*.

In addition to the neuroendocrine organs, hormones are released from epithelial endocrine cells located in the midgut and the midgut cecum in decapods (Christie et al. 2007). The AST-C I/III antibody (*n* = 10 lobsters) labeled an extensive array of such cells (Fig. 3) in the posterior portion of the midgut, although no cells were labeled in other regions of the midgut, i.e., the anterior and central portions of the midgut, or in the posterior midgut cecum (*n* = 10 lobsters). In contrast, no AST-C II-like-immunoreactivity was seen in any portion of the midgut (*n* = 10 lobsters).

### The Three Lobster AST-C Peptides Elicit Distinct Effects on Cardiac Output

**Fig. 4.**
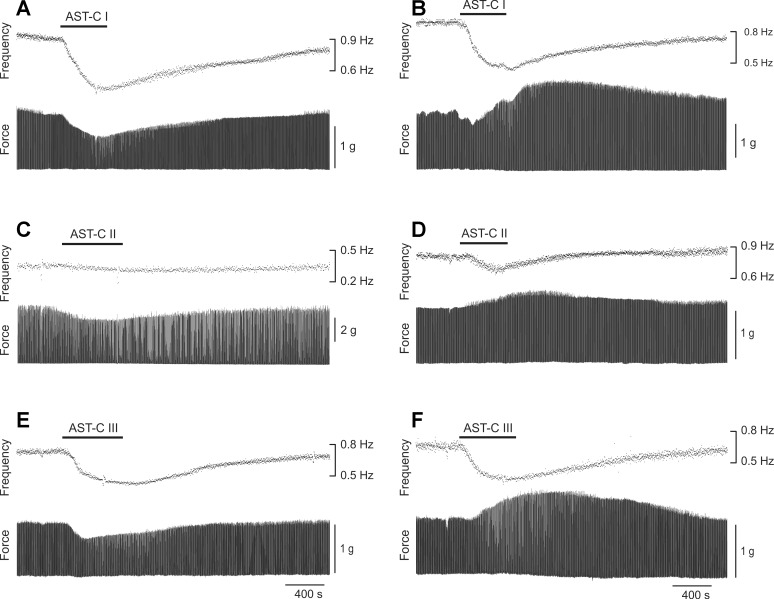
All 3 AST-C peptides can elicit changes in both contraction frequency and amplitude when perfused through an isolated whole heart at 10^−7^ M. *A* and *B*: AST-C I. *C* and *D*: AST-C II. *E* and *F*: AST-C III. Each recording shown is from a different preparation, with the exceptions of *A* and *D*, which are from the same lobster and illustrate the fact that different isoforms can have different effects even within a single animal. All 3 peptides most often elicit decreases in contraction frequency, as shown, but each can elicit either an increase or a decrease in contraction amplitude. Both amplitude and frequency returned approximately to baseline when saline was subsequently perfused through the heart.

**Fig. 5.**
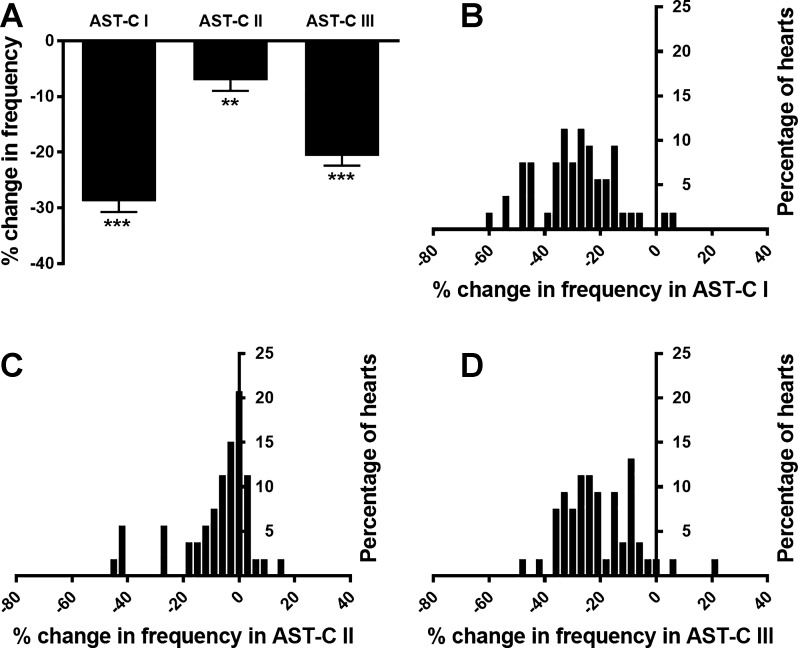
All 3 AST-C peptides elicited a range of changes in contraction frequency, with decreases being predominant. *A*: all 3 AST-C peptides resulted in significant decreases in contraction frequency when perfused through an isolated whole heart at 10^−7^ M. All values are significantly different from a theoretical value of 0; one-sample *t*-test, *n* = 53. **P < 0.01; ***P < 0.0001. Error bars indicate SE. *B*, *C*, and *D*: frequency histograms of the responses to AST-C I, II, and III, respectively. All 3 peptides include both increases and decreases in responses. Data are binned into 3% changes in all cases.

To determine the effects of the three structurally related neuropeptides AST-C I, II, and III on the lobster cardiac neuromuscular system, we perfused each peptide through the isolated whole heart at 10^−7^ M, a concentration within the ranges at which AST-C I and II have previously been shown to exert physiological effects in *H. americanus* (Dickinson et al. 2009; Wiwatpanit et al. 2012). We measured both the heart rate (i.e., contraction frequency) and the force of each heart contraction (i.e., contraction amplitude). When AST-Cs were applied to the heart, a rapid monotonic modulation of contraction cycle frequency generally occurred; these effects washed out when the heart was perfused with fresh saline at the end of the experiment (Fig. 4, *A*–*F*). Although there were instances in which contraction frequency did not change or even showed a small increase, the AST-C peptides most often caused a decrease in contraction frequency; thus the average change in frequency was a significant decrease (one-sample *t*-test with hypothetical value of 0) rather than an increase in all three peptides (Fig. 5*A*; AST-C I: −28.8 ± 1.9%, *P* < 0.0001; AST-C II: −7.1 ± 1.9%, *P* ≤ 0.0005; AST-C III: −20.7 ± 1.8%, *P* < 0.0001; means ± SE; *n* = 53).

**Fig. 6.**
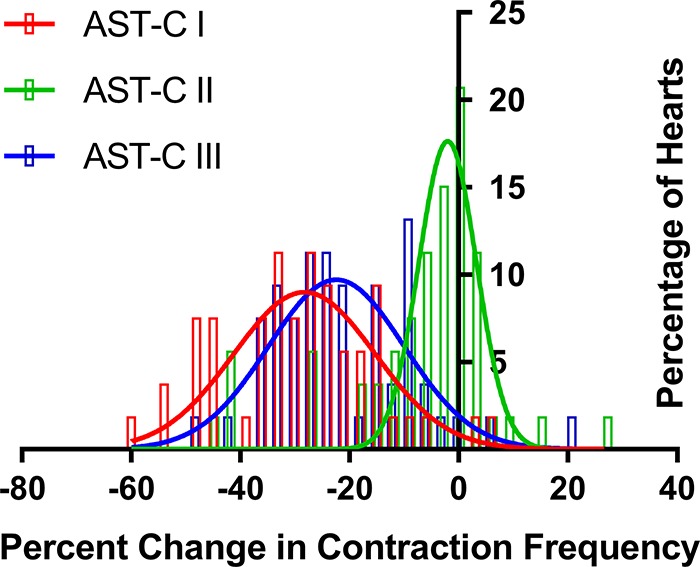
The distributions of changes in frequency elicited by all 3 AST-C peptides could be fit with Gaussian distributions (*r*^2^ = 0.66 for AST-C I, *r*^2^ = 0.81 for AST-C II, and *r*^2^ = 0.67 for AST-C III). The distributions of responses to AST-C I and AST-C III did not differ significantly from one another (Kolmogorov-Smirnov with Bonferroni correction for multiple comparisons, *P* = 0.396), but both differed from the distribution of responses evoked by AST-C II (Kolmogorov-Smirnov with Bonferroni correction for multiple comparisons, *P* < 0.001 for both cases); *n* = 31 bins of 3% each for fits and comparisons of distributions.

To compare the effects of the three AST-Cs relative to one another on contraction frequency, we first examined the distribution of the changes in contraction frequency elicited by each AST-C (Fig. 5, *B*–*D*). Although each of the peptides elicited a range of responses, all of the responses fell into a single distribution, which could be fit with a Gaussian distribution (*r*^2^ = 0.66 for AST-C I, *r*^2^ = 0.81 for AST-C II, and *r*^2^ = 0.67 for AST-C III; Fig. 6). Because the distributions of AST-C I and AST-C III appeared to be much more similar to one another, both in the width and in the mode of the distribution, than they are to the distribution of responses to AST-C II, we used a Kolmogorov-Smirnov nonparametric test (with Bonferroni corrections for multiple comparisons) to compare the distributions of the changes in contraction frequency. We found that the distribution of changes in frequency to AST-C I did not differ significantly from the distribution of changes in frequency to AST-C III (*P* = 0.396). In contrast, the distribution of changes in frequency evoked by perfusion with AST-C II was significantly different from the distributions of changes in frequency elicited by either AST-C I or AST-C III (*P* < 0.001 for both AST-C I vs. AST-C II and AST-C II vs. AST-C III; Fig. 6).

**Fig. 7.**
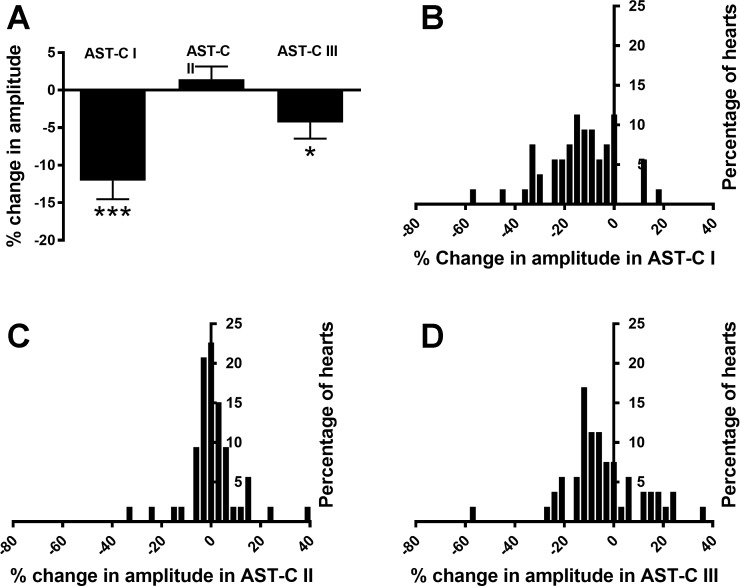
All 3 AST-C peptides elicit a range of changes in contraction amplitude. *A*: when perfused through an isolated whole heart at 10^−7^ M, AST-C I and AST-C III caused, on average, a significant decrease in frequency. AST-C II elicited no significant overall change in amplitude (one-sample *t*-test, *n* = 53). *P < 0.05; ***P < 0.0001. Error bars indicate SE. *B*, *C*, and *D*: frequency histograms of the changes in amplitude recorded in response to AST-C I, II, and III, respectively. All 3 peptides include both increases and decreases in responses. Data are binned by 3% changes in all cases.

**Fig. 8.**
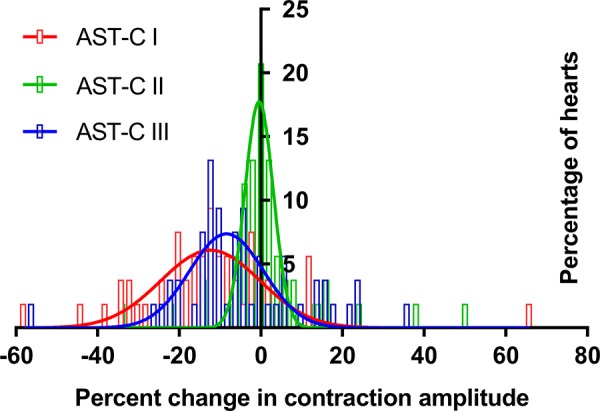
The distributions of changes in amplitude elicited by all 3 AST-C peptides could be fit with Gaussian distributions (*r*^2^ = 0.66 for AST-C I, *r*^2^ = 0.90 for AST-C II, and *r*^2^ = 0.57 for AST-C III). The distributions of responses to all 3 peptides differed from one another (Kolmogorov-Smirnov with Bonferroni corrections for multiple comparisons, *P* < 0.001 for AST-C I vs. AST-C II and AST-C II vs. AST-C III, *P* < 0.05 for AST-C I vs. AST-C III); *n* = 63 bins of 3% each for fits and comparisons of distributions.

Each of the peptides elicited a range of changes in contraction amplitude; for all peptides, this included both increases and decreases in contraction amplitude. Among the preparations in which contraction amplitude increased, a few in each peptide showed an initial small decrease before the increase. Like the effects on contraction frequency, decreases in contraction amplitude (*n* = 46/53 in AST-C I, *n* = 30/53 in AST-C II, and *n* = 36/53 in AST-C III) were more common than increases. The mean changes in response to both AST-C I and AST-C III were small decreases (Fig. 7*A*; AST-C I: −12.0 ± 2.5%; one-sample t-test, *P* < 0.0001; AST-C III: −4.3 ± 2.1%, one-sample *t*-test, *P* = 0.048; *n* = 53). The mean change in response to AST-C II was not significantly different from 0 (Fig. 7*A*; 1.4 ± 1.7%, *n* = 53, one-sample *t*-test, *P* = 0.40). For all of the peptides, the responses fell into a single distribution that could be fit with a Gaussian distribution (Fig. 7, *B*–*D*, and Fig. 8; *r*^2^ = 0.66 for AST-C I, *r*^2^ = 0.90 for AST-C II, and *r*^2^ = 0.570 for AST-C III). Like the distributions of changes in frequency, the distributions of changes in amplitude in response to AST-C I and AST- C III appeared to be more similar to one another than to the distribution of responses to AST-C II. However, the width of the AST-C I distribution appears to be greater than that of the AST-C III distribution, and the mode of the AST-C I distribution is slightly more negative than that of the AST-C III distribution. These differences, taken together, are sufficient to distinguish the two distributions. Consequently, the distributions of the responses to each of the peptides differed significantly from the changes provoked by either of the other two AST-C peptides (Fig. 8; Kolmogorov-Smirnov test with Bonferroni corrections for multiple comparisons: *P* < 0.001 for AST-C I vs. AST-C II and AST-C II vs. AST-C III; *P* = 0.026 for AST-C I vs. AST-C III).

Together, these data suggest that the responses to AST-C I and AST-C III are more similar to one another than either is to the response elicited by AST-C II. Specifically, frequency distributions of the responses to AST-C I and AST-C III are not significantly different, whereas they both differ from the distribution of responses to AST-C II. Moreover, the average changes in contraction amplitude elicited by both AST-C I and III are negative, whereas the average change in amplitude resulting from perfusion with AST-C II is not significantly different from 0. Given these similarities, we decided to examine the extent to which these factors were correlated within individual lobsters.

**Fig. 9.**
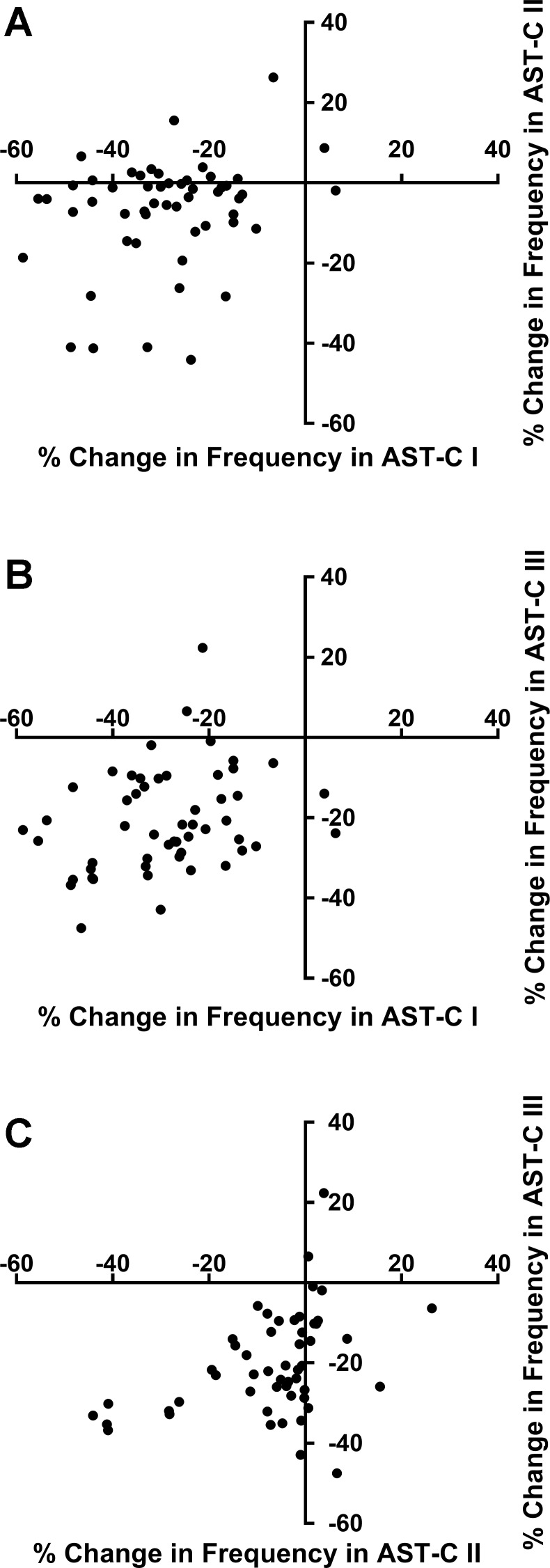
The changes in contraction frequency elicited by AST-C I and AST- III were weakly correlated with one another, as were the changes in frequency elicited by AST-C II and AST-C III. However, in both cases, correlation coefficients are low (<0.2) and significant scatter exists. No significant correlation was seen between the changes in contraction frequency elicited by AST-C I and AST-C II. Graphs of the responses of the same individuals to each pair of peptides are graphed, and fitted with a linear correlation; *n* = 53 hearts. *A*: AST-C I vs. AST-C II (Pearson correlation, *r*^2^ = 0.068, *P* = 0.059). *B*: AST-C I vs. AST-C III (Pearson correlation, *r*^2^ = 0.086, *P* = 0.033). *C*: AST-C II vs. AST-C III (Pearson correlation, *r*^2^ = 0.156, *P* = 0.003).

**Fig. 10.**
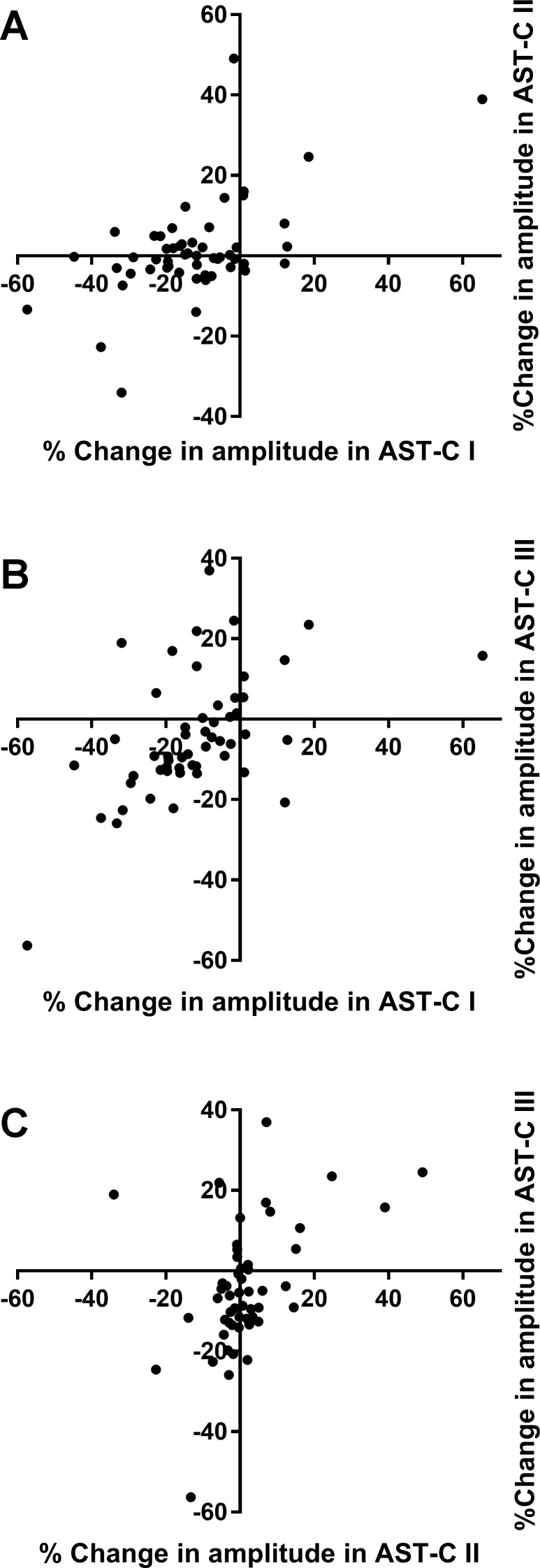
The change in amplitude elicited by perfusion of each of the AST-C isoforms through the heart is correlated to the change elicited in the same heart by perfusion with either of the other two isoforms. However, correlation coefficients (*r*^2^) are all <0.35, and the data all show noticeable scatter; *n* = 53 hearts. *A*: AST-C I vs. AST-C II (Pearson correlation, *r*^2^ = 0.345, *P* = 0.00004). *B*: AST-C I vs. AST-C III (Pearson correlation, *r*^2^ = 0.275, *P* = 0.00006). *C*: AST-C II vs. AST-C III (Pearson correlation, *r*^2^ = 0.196, *P* = 0.00096).

Interestingly, the changes in frequency elicited by the different AST-C peptides were weakly correlated at best (Fig. 9). The changes in frequency resulting from perfusion with AST-C I and AST-C II were uncorrelated (Pearson correlation; *r*^2^ = 0.068, *P* = 0.059; Fig. 9*A*). Moreover, although the percent changes in frequency between AST-C I and AST-C III and between AST-C II and AST-C III were statistically correlated (AST-C I vs. AST-C III, *P* = 0.033, Fig. 9*B*; AST-C II vs. AST-C III, *P* = 0.003, Fig. 9*C*), the correlation coefficients for both regressions were very low (AST-C I vs. AST-C III, *r*^2^ = 0.086; AST-C II vs. AST-C III, *r*^2^ = 0.156). Although the correlations between changes in amplitude were stronger than those for changes in frequency, and all three pairs were significantly correlated, all pairs of correlations were still relatively weak; all correlation coefficients were <0.35, and the data points show considerable scatter (AST-C I vs. AST-C II: *r*^2^ = 0.345, *P* = 0.00004, Fig. 10*A*; AST-C I vs. AST-C II: *r*^2^ = 0.275, *P* = 0.00006, Fig. 10*B*; AST-C II vs. AST-C III: *r*^2^ = 0.196, *P* = 0.00096, Fig. 10*C*; *n* = 53). Interestingly, it is clear that any pair of peptides can cause opposite effects in the same heart (Fig. 10). This is particularly clear for the correlation between change in amplitude in AST-C I vs. AST-C III (Fig. 10*B*); as shown, although the majority of the points appear to be positively correlated, there is a cluster of seven preparations in which AST-C I had little effect or caused a decrease in contraction amplitude, whereas AST-C III perfusion elicited an increase in amplitude.

### Are Effects of the AST-Cs State Dependent?

**Fig. 11.**
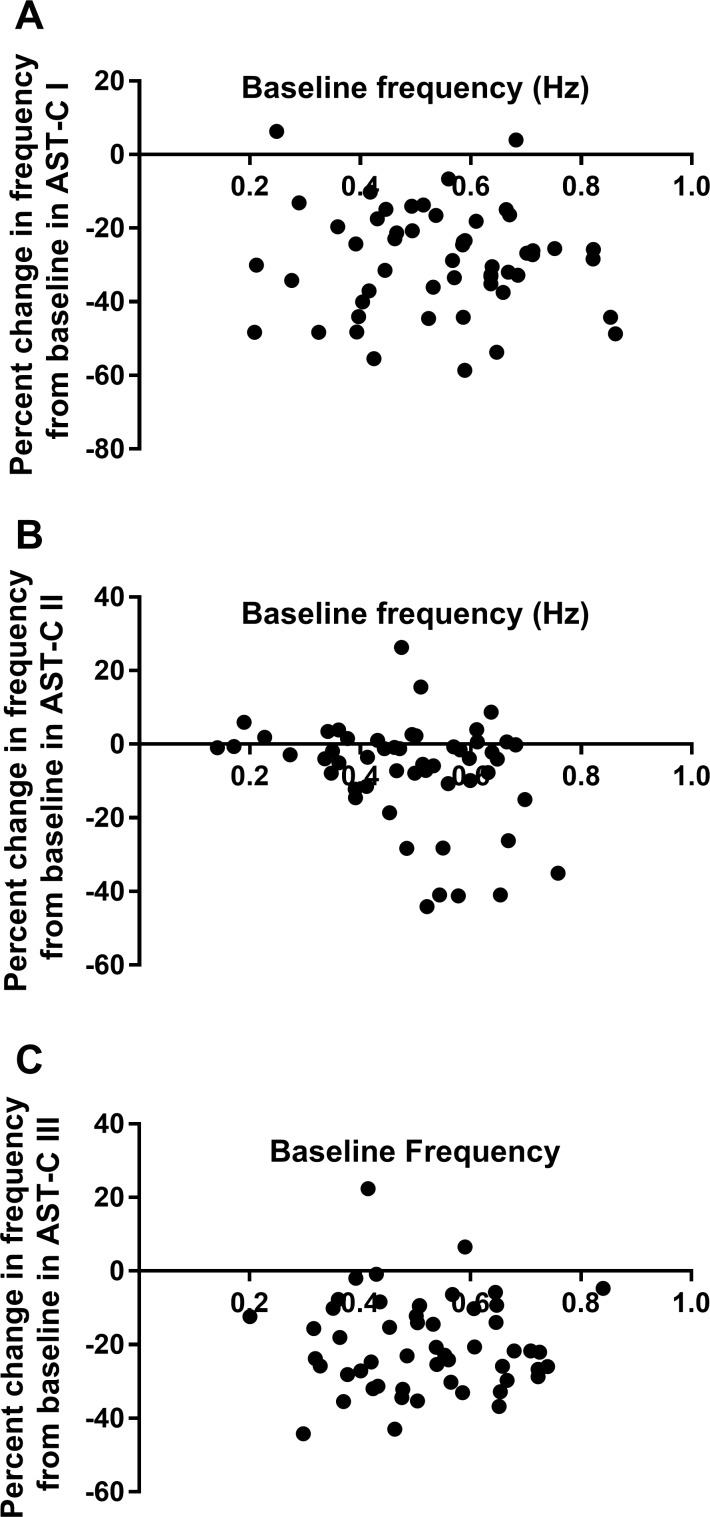
Baseline frequency is not a strong determinant of the change in frequency that is elicited by perfusion with any of the AST-C isoforms. Only for AST-C II is there a significant relationship between baseline frequency and change in frequency during peptide perfusion. Data points were fit with a linear regression; *n* = 53 for all peptide isoforms. *A*: AST-C I (*r*^2^ = 0.004, *P* = 0.659). *B*: AST-C II (*r*^2^ = 0.083, *P* = 0.037). *C*: AST-C III (*r*^2^ = 0.0003, *P* = 0.897).

**Fig. 12.**
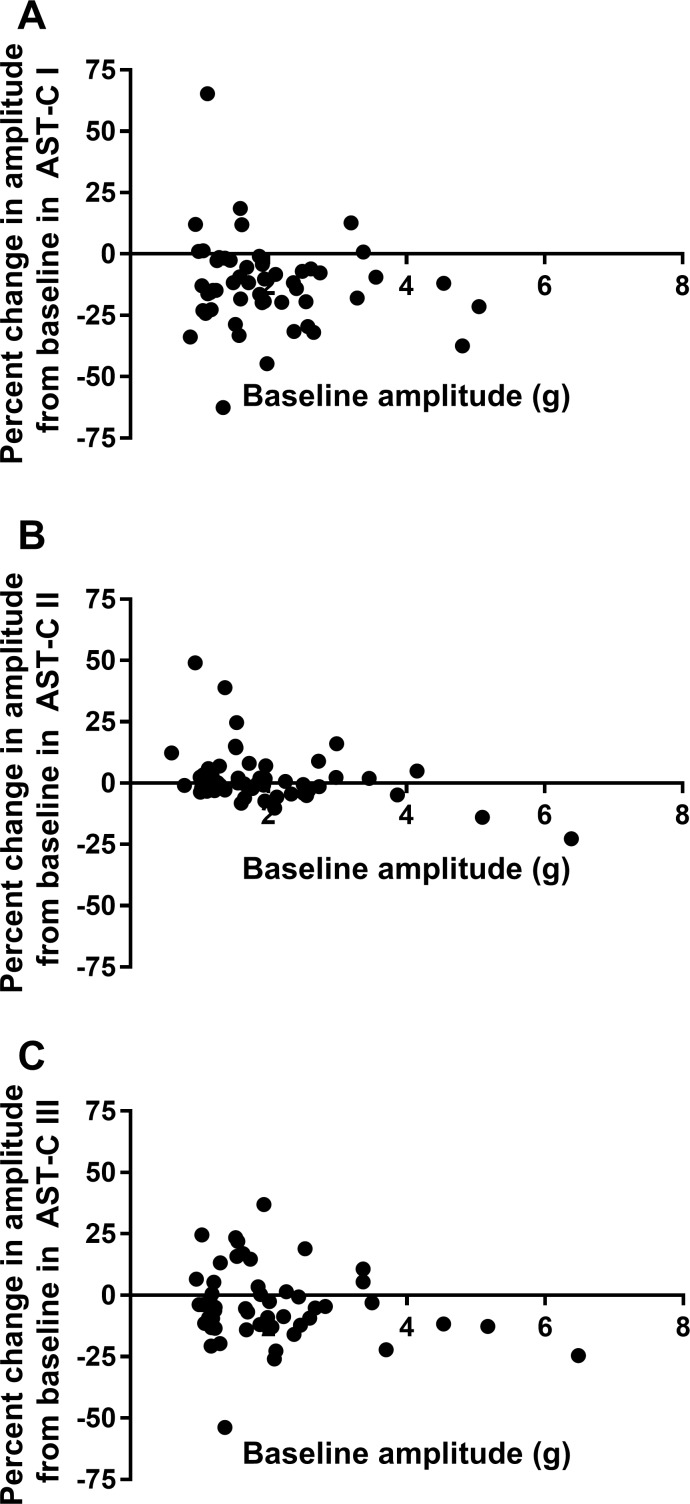
Baseline contraction amplitude is not a strong determinant of the change in amplitude that is elicited by perfusion with any of the AST-C isoforms. Only for AST-C II is there a significant relationship between baseline amplitude and change in amplitude during peptide perfusion. Data points were fit with a linear regression; *n* = 53 for all peptide isoforms. *A*: AST-C I (*r*^2^ = 0.015, *P* = 0.378). *B*: AST-C II (*r*^2^ = 0.145, *P* = 0.005). *C*: AST-C III (*r*^2^ = 0.0323, *P* = 0.198).

Because the effects of many modulators are state dependent, and because the effects of the AST-Cs, particularly AST-C I and AST-C III, are highly variable among preparations, we asked whether the effects of each peptide in a particular heart were dependent on the baseline activity in that heart. We thus looked at the relationship between baseline frequency and percent change in frequency for each of the peptides (Fig. 11) and between baseline contraction amplitude and percent change in contraction amplitude (Fig. 12). There was no significant dependence of change in frequency on baseline frequency for either AST-C I or AST-C III (Fig. 11, *A* and *C*). Similarly, change in amplitude was not dependent on baseline amplitude for these two peptides (Fig. 12, *A* and *C*). Interestingly, as can be seen in Figs. 11*B* and 12*B*, changes in both frequency and amplitude elicited in response to perfusion with AST-C II are to some extent dependent on the baseline parameters (linear regression: *P* = 0.0365 for frequency, *P* = 0.0049 for amplitude). However, although the slopes of the linear fits are significantly different from zero, it is clear from the extent of the scatter and consequent abysmal goodness of fit (*r*^2^ = 0.08 for frequency; *r*^2^ = 0.15 for amplitude) that factors other than baseline are likely more important determinants of the responses to AST-C II, as they are for AST-C I and AST-C III.

## DISCUSSION

### AST-C I/III and AST-C II Are Differentially Distributed Within the Lobster Nervous System and Endocrine/Neuroendocrine Organs

Although both AST-C I and AST-C II have been detected in neural tissues from a large number of decapod species (Stemmler et al. 2010), before this study, no information was available on the cellular distribution of either peptide in the nervous system of any decapod crustacean, let alone the extent to which they might be colocalized in neurons. AST-C III has only recently been identified in the crustaceans (Christie et al. 2017; Veenstra 2015, 2016b). Although it is present in at least seven species of decapods (Veenstra 2016b), neither the extent to which it is conserved across a larger number of species nor the distribution within any given species is known. In this study, we used two antibodies that are specific for subsets of the three AST-C isoforms present in the lobster, *H. americanus* (Christie et al. 2018). One antibody recognized AST-C II exclusively; the other recognized both AST-C I and AST-C III, but not AST-C II. With the use of these reagents in whole mount immunohistochemical experiments, the distributions of AST-C I/III and AST-C II were mapped both in the lobster CG, where at least AST-C I (Wiwatpanit et al. 2012) and AST-C II (Dickinson et al. 2009) are known to be bioactive, and in potential neuroendocrine and endocrine release sites for these peptides.

Although both antibodies labeled some apparent release sites within the PO, AST-C I/III- like immunoreactivity was much more extensive than was AST-C II-like immunoreactivity. With the use of single antibodies, it was impossible to determine the extent of colocalization of AST-C I/III and AST-C II on the basis of morphology, because the profiles labeled by each antibody were indistinguishable. Thus double labeling was conducted, pairing the guinea pig polyclonal AST-C II antibody with one generated against AST-C I in rabbit. The results of these studies suggest that no colocalization of AST-C I/III and AST-C II is present in the PO. Moreover, in both the CG and the endocrine cells of the posterior midgut epithelium, only AST-C I/III was detected. These same antibodies have been used in the lobster STNS to examine the distribution of AST-C peptides in that part of the nervous system (Christie et al. 2018). That study found colocalization of AST-C I/III and AST-C II in only one neuronal soma in each commissural ganglion. Thus, in at least the lobster CG, PO, midgut, and STNS, it appears that the gene encoding AST-C II is expressed largely in populations of cells distinct from those encoding AST-C I and/or AST-C III. Because the antibodies currently available are unable to distinguish between AST-C I and AST-C III, we cannot determine the relative distributions of these two AST-C isoforms. However, if distributions are similar to those in other decapods, we would predict that the AST-C I/III staining we observed in the midgut reflects the presence of AST-C I, and not AST-C III; examination of tissue-specific transcriptomes from *Carcinus maenus* found mRNA encoding AST-C I, but neither AST-C II nor AST-C III, in a midgut-specific transcriptome (Veenstra 2016a). All three AST-C isoforms were found in a mixed nervous system transcriptome in that study, so we are still unable to determine whether AST-C I and III are differentially distributed in the PO, which emanates from the thoracic ganglion.

Previous work from many laboratories has shown that most decapod neuropeptides are present both in regions of central neuropil, e.g., the neuropil of the one or more ganglia, and in one or more neuroendocrine structures, such as the XO-SG and/or the PO (e.g., Christie et al. 1995; Marder et al. 1995). Such dual tissue distribution suggests that these peptides likely function both as locally released paracrines and as circulating hormones in these animals. AST-C I/III-like immunoreactivity was detected in the present study in the lobster CG, as well as in the endocrine cells of the midgut epithelium and in the PO. In contrast, AST-C-II was detected only in the PO; in that tissue, AST-C II staining was less common and less dense than staining for AST-C I/III. Together, these findings suggest that AST-C I and/or III can function as both local and hormonal modulators in the lobster heart; in contrast, AST-C II likely is delivered to the heart only through hormonal pathways. Nonetheless, it is likely that AST-C II also functions as a locally released modulator in other regions of the nervous system, such as the stomatogastric system, where AST-C II-like immunoreactivity is widespread in both neuronal somata and neuropil (Christie et al. 2018).

### The Three AST-C Isoforms Exert Distinct Modulatory Actions on the Lobster Cardiac Neuromuscular System

Although the three AST-C isoforms are structurally similar and are members of the same peptide family, it is clear from the present data that they are able to exert dissimilar effects on the cardiac neuromuscular system, even within an individual lobster. Moreover, all three peptides are able to elicit either increases or decreases in both contraction frequency and amplitude. Interestingly, our findings indicate that the responses to AST-C I and AST-C III are more similar to one another than they are to the responses to AST-C II. Specifically, the distributions of changes in frequency elicited by AST-C I and III do not differ significantly from one another but both differ significantly from the responses elicited by AST-C II. The changes in contraction amplitude triggered by the three peptide isoforms are more complex. The distributions of responses to AST-C I and III differ significantly from one another as well as from that to AST-C II. Thus, for example, the tail on the side of increases in amplitude is more extensive for AST-C III than it is for AST-C I. Nonetheless, both AST-C I and III elicit, on average, a decrease in contraction amplitude, whereas AST-C II does not, on average, alter contraction amplitude. Finally, although none of the responses are highly dependent on baseline frequency or amplitude, the responses to AST-C II are weakly dependent on baseline parameters, whereas responses to both AST-C I and III appear to be completely independent of this factor.

The distributions of changes in contraction frequency elicited by AST-C I and AST-C III isoforms were very similar and differed from the distribution of responses to AST-C II (Fig. 9). We thus predicted that the responses of individual lobsters to AST-C I and AST-C III would be correlated but that these responses would not be correlated with the responses to AST-C II. Surprisingly, the changes in frequency seen in response to AST-C I and AST-C III within individual lobsters were not more highly correlated than were the responses to AST-C I and AST-C II. In fact, the correlation coefficients between the changes in frequency elicited by all three pairs of peptides (i.e., AST-C I vs. ASTC II, AST-C I vs. AST-C III, and AST-C II vs. AST-C III) are very low. Thus, despite the similar distributions of the responses to AST-C I and AST-C III in the total population of lobsters we examined, the extent of the variance that is explained by this correlation within any individual lobster is quite small.

A difference between population and individual patterns of responses was also seen in the changes in amplitude elicited by the three peptide isoforms. Thus, although the distribution of responses elicited by each of the AST-C isoforms differs from that elicited by the other two isoforms, all of the combinations are somewhat correlated within individual lobsters. However, although the slope of each of these correlations is significantly non-zero, all three correlation coefficients are very low. Surprisingly, the changes in amplitude are more highly correlated within lobsters than are the changes in frequency, even though the distributions of amplitude changes elicited by the three peptides all differ significantly from one another.

Previous work has shown that AST-C I has differential effects on contraction amplitude among lobsters (Wiwatpanit et al. 2012); in this study we found that the same is true for the other AST-C isoforms that are native to *H. americanus*. Moreover, we found that the three peptides can exert differential effects even within a single lobster; for example, one peptide can elicit an increase in contraction amplitude, whereas the other two result in contraction amplitude decreases (e.g., compare data in Fig. 4, *A* and *D*, which are taken from the same lobster). This suggests that there is at least one receptor that differentiates between these peptides, but it does not preclude the possibility that there are additional receptors that are activated by two or all three peptides. In fact, a recent study identified three putative AST-C receptors in an *H. americanus* mixed neural tissue transcriptome (Christie et al. 2015). The relative binding efficacies of these receptors for the three peptides are currently unknown but will be important in understanding the mechanisms that underlie these differential responses. It will also be important to determine which of these receptors are expressed in the CG, including whether they are differentially expressed in hearts that show different responses to the three peptides. Interestingly, the distributions of effects on contraction frequency between AST-C I and III were more similar than those between AST-C II and either of the other two peptides. Given the fact that AST-C II is COOH-terminally amidated, whereas neither AST-C I nor AST-C III is amidated, it is possible that the COOH-terminal amidation is important in determining binding to the different AST-C receptors. Interestingly, a large proportion of known neuropeptides require an amidated COOH-terminal to be biologically active (Eipper et al. 1992; Merkler 1994). For example, crustacean hyperglycemic hormone (CHH) in crayfish is inactive when the COOH-terminal amidation is removed (Mosco et al. 2008). In the case of AST-C, the amide is clearly not required for biological activity, because the nonamidated AST-C I and III exert effects that are, on average, greater than those of the amidated AST-C II. This may be a characteristic of C-type allatostatins; in *Manduca sext*a, the effects of a synthetically amidated version of the native peptide did not differ from the effects of the free acid form of the same peptide (Kramer et al. 1991). However, the amide could well be important in determining binding to specific receptors, and thus in determining the specific responses seen. Interestingly, the antibodies that we developed to detect C-type allatostatins show a similar selectivity: one binds specifically to AST-C II, whereas the other does not recognize AST-C II but binds to both AST-C I and AST-C III.

Because the lobster heart is neurogenic, and peptides in these experiments were applied to the whole heart, there are a number of sites at which these peptides could have exerted their effects. The lobster neuromuscular system used in these experiments included the pattern generator in the CG, which generates the rhythmic output that drives cardiac muscular contraction, as well as the neuromuscular junction, the cardiac muscles themselves, and two known feedback systems (Cooke 2002; García-Crescioni et al. 2010; García-Crescioni and Miller 2011; Mahadevan et al. 2004). Because of the complex nature of the feedback from the periphery to the CG, it is possible that changes at any of these levels could be responsible for the observed alterations of cardiac activity. Moreover, detailed studies of the mechanisms responsible for the modulation by other peptides, including myosuppressin (Stevens et al. 2009), crustacean cardioactive peptide (CCAP; Fort et al. 2007b), and several FMRFamide-like peptides (Dickinson et al. 2015a; Fort et al. 2007a), have shown that the effects of a given modulator on the whole heart do not directly reflect the effects on the CG and the periphery, because the alterations of contraction in turn alter the feedback to the pattern generator. However, Wiwatpanit et al. (2012) showed that the differential responses to AST-C I were due entirely to effects on the CG; this peptide does not alter contraction amplitude when applied to a preparation in which the motor output is prevented from changing in response to AST-C I by removing the CG and instead stimulating the motor nerves with a constant pattern. Whether the same is true for AST-C II and III remains to be tested.

### Potential Mechanisms Underlying the Differential Effects of AST-C Isoforms

Although most neuromodulators that have been examined elicit effects on a given neural network that are qualitatively similar across individuals, this is not always the case. For example, serotonin applied to the pyloric pattern generator in spiny lobsters elicits an array of responses on cycle frequency, including increases, decreases, and no effect (Spitzer et al. 2008). In the crab, octopamine can elicit either a decrease or an increase in presynaptic transmission onto the claw closer muscles (Djokaj et al. 2001). Similarly, serotonin can either potentiate or decrease the response of the lateral giant neuron to sensory stimulation in the crayfish, an effect that is dependent on the social status of the animal (Yeh et al. 1997). At least some such differences can be attributed to metamodulation (Edwards et al. 2002; Mesce 2002), in which other modulators are responsible for altering the response to a given modulator across time, even within the same individual.

The effects of many other modulators differ quantitatively across individuals, even though these effects may be qualitatively similar. A number of previous studies have found that the differential effects of such neuromodulators can result from differences in previous activity, physiological state, or the baseline activity of the modulated network (e.g., Birmingham et al. 2003; Rodgers et al. 2011; reviewed in Marder et al. 2014). For example, Nusbaum and Marder (1989) described activity-dependent modulation by the peptide proctolin in the crab (*C. borealis*) stomatogastric system; the extent of modulation of the pyloric motor pattern was strongly dependent on the baseline conditions, with preparations in which baseline activity was low showing much larger increases in cycle frequency than preparations in which activity was initially strong. Nagy and Dickinson (1983) similarly showed that the activity of an identified modulatory neuron activated the pyloric network strongly in those preparations in which baseline activity was low, but had little effect when the network was already strongly active. Functionally, in these systems, this type of activity dependence leads to similar motor outputs when the modulator is present or active (Nagy and Dickinson 1983). However, a similar pattern of dependence on baseline activity is present in the crustacean STNS in response to several allatostatin peptides, including A-, B-, and C-type allatostatins (Fu et al. 2007; Ma et al. 2009; Skiebe and Schneider 1994; Szabo et al. 2011). Although these three groups of peptides are unrelated, they all exert inhibitory effects; thus the less active preparations are more inhibited so that the effect is minimal on active preparations but exaggerated on less active preparations. Instead of leading to similar activity across preparations, the differences in the level of motor output in these preparations are amplified.

Although the functional effects of inhibitory and excitatory modulators may not follow the same pattern when these effects are dependent on baseline activity, they can be explained by a similar mechanism. Specifically, when the pattern generator is more active, average ionic conductances in pattern generator neurons are higher; the resulting lower input resistance will result in decreased overall effects of other conductances, including any that are activated by the neuromodulators. In the case of the effects of the AST-Cs on the lobster heart, however, there is little dependence on baseline activity. It is possible that baseline activity, via a mechanism such as average baseline conductance, is contributing slightly to the effects of AST-C II, but it appears likely to be uninvolved in the variability of the responses to AST-C I or AST-C III, which show no dependence on baseline activity. Moreover, the fact that the different AST-C peptides can exert very different effects, both quantitatively and qualitatively, in some lobsters, but similar ones in others, suggests that the baseline activity is not a key determinant of the effects of these peptides.

A number of studies have now shown that a given activity pattern can result from the activation of multiple different arrays of channel conductances within neurons (Ball et al. 2010; Goaillard et al. 2009; Prinz et al. 2004; Taylor et al. 2009). Specifically, it is clear from both studies in which conductances are perturbed and those in which conductances and/or the levels of mRNA encoding ionic channels have been measured (e.g., Ransdell et al. 2012, 2013; Schulz et al. 2006, 2007; Swensen and Bean 2005) that an appropriate balance of particular arrays of conductances is important in enabling neurons to generate the same output. One such system in which it is clear that similar output can be generated with different arrays of channels is the crustacean CG (Ransdell et al. 2013). This leads to the possibility that lobster cardiac neurons expressing different sets of conductances might respond differently to the same neuromodulator, thus generating differential responses among individuals.

On another level, the actual movements of the heart, which were measured in this study, are a function not only of the output of the CG but also of the nonlinear neuromuscular transform (Brezina et al. 2000a, 2000b; Williams et al. 2013). Measurements of the neuromuscular transform in the lobster heart have shown that contractions of a given amplitude can be induced by multiple CG outputs, particularly combinations of duty cycle and burst frequency; thus even similar changes in bursting parameters can elicit different changes in contraction amplitude. In fact, much of the variability in the responses of the lobster heart to AST-C I can be explained by the neuromuscular transform (Williams et al. 2013). Notably, when the cycle frequency of cardiac bursts decreases but burst duration remains constant, duty cycle decreases, which can lead to a decreased contraction amplitude (Wiwatpanit et al. 2012). A similar decrease in cycle frequency accompanied by an increase in burst duration can result in an increase in contraction amplitude (Williams et al. 2013). The decrease in cycle frequency often precedes the increase in burst duration, resulting in a mixed effect in which the contraction amplitude first decreases and then increases (Wiwatpanit et al. 2012). Whether the effects of the AST-Cs on cycle frequency and burst duration are mediated by the same receptors at different peptide concentrations or whether they are mediated by different receptors, which have either different time courses or concentration dependencies, is not currently known but would be a fruitful subject of future studies.

Nonetheless, although these mechanisms could account for variability in the response to a single AST-C isoform across individuals, they are less able to explain the variability in the relative responses of the different AST-C isoforms. One factor that may help to explain this, particularly in combination with the other factors discussed above, is the possible existence of multiple AST-C receptors. Recent transcriptomic studies have identified three putative AST-C receptors in the nervous system of *H. americanus* (Christie et al. 2015). If these receptors are differentially expressed within the CG and are differentially activated by the three AST-C isoforms, this could underlie the range of responses to the AST-C isoforms that we recorded in the present study. This would in many respects parallel the responses of the pyloric network in the spiny lobster STNS to the modulator serotonin (Spitzer et al. 2008). In this instance, the neuromodulator appears to activate multiple receptors, each of which is associated with a particular response component. Because some of these components mediate increases and others mediate decreases in cycle frequency, the activation of different balances of these opposing components can lead to different responses from the system as a whole (Spitzer et al. 2008).

On a more global level, we do not yet understand the factors that determine the responses to each peptide, nor the extent to which those responses are similar or different among the isoforms. One attractive possibility is metamodulation, as has been shown to explain differential modulation in crayfish (Edwards et al. 2002) and in the leech (Mesce 2002). A previous study (Wiwatpanit et al. 2012) suggested the possibility that molt stage may be one factor contributing to metamodulation in this instance. As was the case for AST-C I in that study, we found in the present study that individuals in which we recorded particular patterns of response, e.g., increased amplitude in response to AST-C III with decreases to AST-C I, were often clustered in time. One hypothesis that we are beginning to test is that molt stage is correlated with receptor expression. However, other possibilities certainly exist.

## GRANTS

Financial support for this study was provided by National Science Foundation Grants IOS-1353023 and IOS-1354567, National Institutes of Health Grants 5P20RR016463-12 and 8P20GM103423-12, the Cades Foundation of Honolulu, HI, and a gift from the Henry L. and Grace Doherty Charitable Foundation to Bowdoin College.

## DISCLOSURES

No conflicts of interest, financial or otherwise, are declared by the authors.

## AUTHOR CONTRIBUTIONS

P.S.D and A.E.C. conceived and designed research; P.S.D., M.K.A., E.S.D., R.F., A.M., S.P., B.W.P., A.P.W., M.E.S., P.J.W., T.W., and A.E.C. performed experiments; P.S.D., M.K.A., E.S.D., S.P., B.W.P., A.P.W., M.E.S., P.J.W., and T.W. analyzed data; P.S.D., E.S.D., R.F., A.M., S.P., B.W.P., A.P.W., M.E.S., P.J.W., T.W., and A.E.C. interpreted results of experiments; P.S.D., E.S.D., and A.E.C. prepared figures; P.S.D drafted manuscript; P.S.D., M.K.A., E.S.D., R.F., A.M., S.P., B.W.P., A.P.W., M.E.S., P.J.W., T.W., and A.E.C. edited and revised manuscript; P.S.D., M.K.A., E.S.D., A.M., S.P., B.W.P., A.P.W., M.E.S., P.J.W., T.W., and A.E.C. approved final version of manuscript.
